# B4 suppresses lymphoma progression by inhibiting fibroblast growth factor binding protein 1 through intrinsic apoptosis

**DOI:** 10.3389/fphar.2024.1408389

**Published:** 2024-06-28

**Authors:** Krishnapriya M. Varier, Gou Dan, Xiaolong Li, Wuling Liu, Fei Jiang, Ke-Gang Linghu, Yanmei Li, Yaacov Ben-David, Nenling Zhang, Chaoda Xiao, Babu Gajendran, Xiangchun Shen

**Affiliations:** ^1^ School of Pharmaceutical Sciences, Guizhou Medical University, Guiyang, China; ^2^ State Key Laboratory for Functions and Applications of Medicinal Plants, Guizhou Medical University, Guiyang, China

**Keywords:** lymphoma, flavonoid, Fgf signaling, FGFBP1, inhibitor, apoptosis

## Abstract

Lymphoma positions as the fifth most common cancer, in the world, reporting remarkable deaths every year. Several promising strategies to counter this disease recently include utilizing small molecules that specifically target the lymphoma cellular proteins to overwhelm its progression. FGFBP1 is a soluble intracellular protein that progresses cancer cell proliferation and is upregulated in several cancers. Therefore, inhibiting FGFBP1 could significantly slow down lymphoma progression through triggering apoptosis. Thus, in this study, a flavonoid B4, isolated from *Cajanus cajan*, has been investigated for its effects of B4 on lymphoma, specifically as an FGFBP1 inhibitor. B4 could selectively hinder the growth of lymphoma cells by inducing caspase-dependent intrinsic apoptosis through G_1_/S transition phase cell cycle arrest. RNA sequencing analysis revealed that B4 regulates the genes involved in B-cell proliferation and DNA replication by inhibiting FGFBP1 *in vitro*. B4 increases the survival rate of lymphoma mice. B4 also represses the growth of patient-derived primary lymphoma cells through FGFBP1 inhibition. Drug affinity responsive target stability experimentations authorize that B4 powerfully binds to FGFBP1. The overexpression of FGFBP1 raises the pharmacological sensitivity of B4, supplementing its specific action on lymphoma cells. This study pioneers the estimation of B4 as a possible anticancer agent for lymphoma treatment. These outcomes highlight its selective inhibitory effects on lymphoma cell growth by downregulating FGFBP1 expression through intrinsic apoptosis, causing mitochondrial and DNA damage, ultimately leading to the inhibition of lymphoma progression. These suggest B4 may be a novel FGFBP1 inhibitor for the lymphoma treatment.

## 1 Introduction

Lymphoma is an ailment considered for the uninhibited proliferation of lymphoid cells, originating from more mature B or T cells. This neoplastic state comprehends around 100 different tumors of the blood-forming system and is categorized by the irregular expansion of lymph nodes (Zuo et al., 2021). Globally, lymphoma ranks as the fifth most common cancer, with approximately 544,000 new cases and 260,000 deaths reported each year. Unfortunately, despite advancements in treatment schedules, the mortality rates associated with the disease have shown substantial advances over the past five decades (Milano, 2023). Therefore, it needs to explore newfangled therapies that effectively target lymphoma and could selectively induce apoptosis in these cells to combat the disease effectively.

Apoptosis or programmed cell death of cells initiates an intrinsic mechanism of self-destruction to maintain tissue homeostasis. Flaws in these paths can induce apoptosis, leading to the neoplastic extension of cellular populations. Several chemotherapeutics practice small molecules that can encourage apoptosis, by intrinsic or extrinsic methods (Zhou et al., 2023). This process involves the activation of proapoptotic marker molecules such as Bax, Cytochrome C (Cyt. C), and cyclins, as well as anti-apoptotic markers like Bcl-2. In the intrinsic pathway, the mitochondria will eventually release Cyt. C into the cytosol, initiating the activation of Caspase 3 and 9 (Ma et al., 2023). However, apoptosis through the mitochondria can be inhibited at diverse levels by anti-apoptotic proteins, including the Bcl-2 family and apoptosis inhibitor proteins (Sarosiek and Wood, 2023). The proapoptotic Bcl-2 family protein Bax mediates these signals, leading to PARP1 cleavage and amendments in cell cycle checkpoint markers like Cyclin D1, ultimately resulting in mitochondrial damage and cell death. On the other hand, the extrinsic pathway relies on the direct activation of death receptors, causing DNA damage (Zhang et al., 2023). These apoptotic pathways also involve numerous signaling molecules that can suppress cancer cell growth and induce intrinsic or extrinsic apoptosis in different cancers (Ma et al., 2023). Thus, understanding the intricate mechanisms of apoptosis and its regulation by apoptotic initiators like secreted chaperones is essential for developing effective therapeutic strategies for cancer treatment.

Fibroblast growth factor-binding protein 1 (FGFBP1) is a secreted chaperone that mobilizes paracrine-acting fibroblast growth factors (FGFs) from the extracellular matrix and presents them to their respective receptors. FGFBP1 amplifies FGF signaling pathways thereby promoting angiogenesis, a crucial course in cancer advancement. This gene is found to be significantly upregulated in numerous cancer types, including squamous cell carcinoma, melanoma, pancreatic and colon cancer (Tassi et al., 2011; Wang et al., 2021; Zhang et al., 2021; Qiu et al., 2023). Studies have shown that the knockdown of FGFBP1 can induce apoptosis in colon carcinoma cells mediated through caspase-3/9 activation and alterations in mitochondrial metabolism upon FGFBP1 knockdown. These effects are based on the upregulation of Bax and the downregulation of Bcl-2. The observed outcomes stem from the activation of Bax and the suppression of Bcl-2 (Schulze et al., 2011). However, the therapeutic potential of obstructing FGFBP1 by small molecules or bioactive phytochemicals is a novel strategy in lymphoma therapeutics.

Phytochemicals, particularly flavonoids, have been expansively studied for their expanded anticancer properties. Small molecule derived from *Cajanus cajan* leaves and roots have been shown to inhibit the growth of cancer cells through the mitochondria-dependent apoptosis pathway (Varier et al., 2022). Methanol extracts of *C. cajan* are reported to show anti-cancer effects in breast adenocarcinoma (MCF-7), lung carcinoma (COR-L23), and melanoma (C32) cell lines (Ashidi et al., 2010). However, there is currently a lack of studies investigating the potential anti-lymphoma action of 3,5-Dihydroxy-2-(4-hydroxyphenyl)-8,8-dimethyl-2,3-dihydro-4H, 8H-pyrano [2,3-f]chromen-4-one (B4), a natural flavonoid found in *C. cajan* leaves. Therefore, the objective of this study is to explore the potential anti-lymphoma effect of B4, specifically focusing on its ability to inhibit FGFBP1 and trigger caspase-dependent apoptosis.

## 2 Materials and methods

### 2.1 Cell lines, chemicals and reagents

The following cell lines were used in this study: EL4 (Mouse lymphoma; ATCC^®^ TIB-39™), Raji (Human B cell lymphoma; ATCC^®^ CCL-86™), OCI_LY10 (Human B cell lymphoma; RRID, CVCL_8795), CB3 (Mouse erythroleukemia; Generated by us), HEL (Human erythroleukemia; ATCC^®^ TIB-180™), CB7 (Mouse erythroleukemia; Generated by us), K562 (Human chronic myelogenous leukemia; ATCC^®^ CCL243™), DP-17 (Mouse erythroleukemia; Generated by us), MCF-7 (Human breast cancer; ATCC^®^ HTB-22™), MDA-MB-468 (Human breast cancer; HTB-132™), MDA-MB-231 (Human breast cancer; ATCC^®^ HTB-26™), 4T1 (Mouse breast cancer; ATCC^®^ CRL-2539™), WM9 (Human metastatic melanoma; Rockland; WM9-01-0001), HL7702 (Normal human liver cells), and H9C2 (Rat myoblast; ATCC^®^ CRL-1446™).

The reagents and chemicals used in this study are of analytical grade. The antibodies Bax (Pt 60267-1-lg), Bcl-2 (12789-1-AP), Cyclin D1 (26939-1-AP), Caspase3 (19677-1-AP), Caspase 9 (10380-1-AP), PARP1 (13371-1-AP), Cytochrome C (66264-1-lg), FGFBP1 (25006-1-AP), secondary antibody anti-mouse IgG (SA00001-1), and secondary antibody anti-rabbit IgG (SA00001-2) were purchased from Proteintech (US). The antibodies Cleaved-caspase3 (#9661), APAF-1 (#8723), and Cleaved-caspase9 (#9505) were obtained from Cell Signalling Technology (CST, US). The GAPDH (AP0063), and phospho-histone γ-H2AX (#AF3187) and were purchased from Affinity Biosciences (CN) and Bioworld Technology (CN), respectively. The 2-mercaptoethanol (Sigma-Aldrich, US), Penicillin/streptomycin (Gibco), Stem cell factor (SCF; R&D Systems, US), and EPO were obtained for primary cell suspension maintenance. The suspension cells were maintained in RPMI 1640 and DMEM medium (Gibco, US) supplemented with 5% fetal bovine serum (FBS; Biological Industries, IL). Primary cell culture was maintained in alpha MEM (Gibco, US) medium with 15% FBS. All cells were incubated at 37°C with 95% humidity and 5% CO_2_. As previously described, the B4 was isolated and characterized from Cajanus Cajan (Lei et al., 2022).

### 2.2 Cell viability and morphological analysis

The cells were treated with various concentrations of B4 (10, 20, 30, 40, and 50 µM) in triplicates. As a control, the cells were given 0.01% DMSO. After incubating for 24, 48, and 72 h, 20 µL of MTT solution (5 mg/mL) was added to each well, and the cells were further incubated for 4 hours. Subsequently, the cells were centrifuged (4000 RPM) for 10 min. The supernatants were discarded, and 150 µL of DMSO was added. The plates were placed on a shaker for 5 minutes to dissolve the formazan products. The absorbance of the formazan products was measured (490 nm) using the Synergy2 modular Multi-Mode Reader (BioTek, Winooski, VT, United States) and the inhibition rate was calculated using the following equation: Inhibition rate (%) = (OD of treated/OD of control) × 100. The cellular morphology of the DMSO (control) or B4-treated cells was recorded using a Nikon inverted microscope (Gajendran et al., 2020).

### 2.3 Apoptosis analysis

To analyze apoptosis, the DMSO and B4-treated cells (1 × 10^6^ cells/well) were seeded in 6-well plates and incubated at different concentrations for 48, and 72 h. After treatment, the cells were collected and centrifuged, followed by two washes with precooled PBS. The cells were then suspended in 500 µL of binding buffer (1X), 2.5 µL of annexin V-FITC (Becton, Dickinson, US), and 2.5 µL of propidium iodide (Becton, Dickinson, US) and incubated for 20 min at room temperature. Later cells were analyzed using a flow cytometer (ACEA Biosciences, Inc. San Diego, US; Varier et al., 2022).

### 2.4 Cell cycle analysis

In 6-well plates, cells were seeded at a density of 1 × 10^6^ cells/well and treated with different concentrations of B4 for 48 and 72 h. Following treatment, the cells were collected and washed (twice) with precooled PBS. Subsequently, the cells were fixed with 70% ethanol at −20°C and incubated overnight. After washing (with PBS), the cells were incubated in a mixture containing propidium iodide (100 μg/mL), RNase inhibitor (10 μg/mL), and Triton X-100 (0.5 μg/mL) in the dark for 30 min at 37°C. Finally, the samples were analyzed using a flow cytometer (ACEA Biosciences, Inc. San Diego, US; Varier et al., 2022).

### 2.5 Transmission electron microscope (TEM) studies

After treating the OCI_LY10 cells with DMSO and B4 (27.6 µM) for 72 h, they were prefixed using a 3% glutaraldehyde solution. Then, the cells were postfixed with osmium tetroxide (1%), dehydrated using a series of acetone solutions, and infiltrated with Epox 812 resin for an extended period. Finally, the cells were embedded for further analysis. The embedded cells were stained with uranyl acetate and lead citrate (Kim et al., 2018). The sections were examined using a JEM-1400-FLASH TEM from JOEL, US, at the Lilai Biomedicine Experiment Center in Chengdu, China.

### 2.6 Hoechst 33258 staining

In a 6-well plate, cells were incubated at a density of 4 × 10^4^ cells/well and treated with various concentrations of B4 for 48 and 72 h. Following the treatment period, the cells were collected and stained with Hoechst 33258 dye, by the manufacturer instructions (Beyotime, Jiangsu, CN). The morphological changes of the nucleus were then captured using fluorescence microscopy from Leica, DE; Varier et al., 2022).

### 2.7 Detection of MMP and ROS in cells

To assess the variations in mitochondrial membrane potential (MMP), the MMP assay kit from Beyotime (CN) was utilized according to the manufacturer’s protocols. Briefly, the MMP detection was carried out on both DMSO and B4 treated with different concentrations for 72 h. The cells were probed with JC-1 for 30 min after staining. Subsequently, DMI1 fluorescence microscopy from Leica (DE) was employed to analyze the stained cells at the indicated treatment concentrations, with a cellular density of 4 × 10^4^ (Varier et al., 2022).

### 2.8 RNA sequencing analysis and protein-protein interactions prediction

EL4 cells were treated with DMSO and B4 (27.5 µM) for 72 h, after which BGI Genomics (CN) performed RNA sequencing. The RNAseq data was mapped and analyzed using differential expression analysis with Biovinci software (version 1.1.5). The analysis involved mapping the data onto curated gene groups using Ward’s minimum variance and Euclidean distance. The data was subsequently represented through a heatmap [9], utilizing GraphPad Prism 9.0. The FGFBP1-related protein-protein interactions were predicted using the STRING database analysis (Varier et al., 2022).

### 2.9 Gene expression analysis

Cell cultures were maintained in the presence of varying concentrations of B4 or DMSO for 72 h. Following this, total RNA was extracted from the cells using Invitrogen’s TRIzol reagent (Carlsbad, US). An equal amount of RNA (2 μg) was then reverse transcribed into cDNA using the Hifair III 1st Strand cDNA Synthesis Kit from Yeasen (Shanghai, CN). Quantitative real-time PCR was performed using the Heiff UNICON qPCR SYBR Green master mix (Yeasen, CN) on a Bio-Rad (US) Real-time PCR System. The expression of mRNA genes was analyzed using gene-specific primers (Varier et al., 2022). The primers used for the study can be found in Table 1.

**TABLE 1:** The sequences of primers used in RT-qPCR.

Gene	Sense	Antisense	Species
FGF1	TGC​CTC​CAG​GGA​ATT​ACA​AG	TAT​AAA​AGC​CCG​TCG​GTG​TC	Human
FGF2	AGA​GCG​ACC​CTC​ACA​TCA​AG	TCG​TTT​CAG​TGC​CAC​ATA​CC	
FGF3	AGA​TAA​CGG​CAG​TGG​AGG​TG	TTA​TAG​CCC​AGC​TCG​TGG​AT	
FGF7	CAA​ACA​GCG​TCA​CAG​CAA​CT	GTA​GTG​CTC​CGG​GTG​TTC​AT	
FGF10	ATG​TCC​GCT​GGA​GAA​AGC​TA	TTT​CCC​CTT​CTT​GTT​CAT​GG	
FGF22	TCC​TGG​AGA​TCC​GCT​CTG​TA	GTA​GGT​GTT​GTG​GCC​GTT​CT	
FGFR2	CAG​AGA​CCA​ACG​TTC​AAG​CA	GAG​GAA​GGC​ATG​GTT​CGT​AA	
TBX2	GGG​ACC​AGT​TCC​ACA​AGC​TA	GGT​GGA​TGT​ACA​TGC​GTT​TG	
HSPG2	CAC​CTG​ATC​TCC​ACC​CAC​TT	GCA​GCT​GCC​AGT​AGA​AGG​AC	
PROM1	GCA​ATC​TCC​CTG​TTG​GTG​AT	TCA​GAT​CTG​TGA​ACG​CCT​TG	
FGFBP1	CTT​CAC​AGC​AAA​GTG​GTC​TCA	GAC​ACA​GGA​AAA​TTC​ATG​GTC​CA	
GAPDH	CAA​CGA​ATT​TGG​CTA​CAG​CA	AGG​GGT​CTA​CAT​GGC​AAC​TG	
FGF1	AAG​CAT​GGG​ATC​TCG​GAA​CA	GCT​AAC​ATC​CAC​GGC​TGA​AG	Mouse
FGF2	ACT​CCA​AGC​AGA​AGA​GAG​AGG	CGT​TTC​AGT​GCC​ACA​TAC​CA	
FGF3	CCG​CAC​ACA​AAA​GTC​CTC​TC	CAT​GAT​CAC​TTG​GGC​TCT​GC	
FGF7	GCA​AAG​TGA​AAG​GGA​CCC​AG	CAA​CGA​ACA​TTT​CCC​CTC​CG	
FGF10	AGG​CTG​TTC​TCC​TTC​ACC​AA	TCC​CCT​TCT​TGT​TCA​TGG​CT	
FGF22	AAC​GGC​TAC​AAC​ACA​TAC​GC	GCC​CTT​CAA​GAC​GAG​ACC​AA	
FGFR2	TTT​GCC​GAA​TGA​AGA​CCA​CG	CGT​TGT​TAT​CCT​CAC​CAG​CG	
TBX2	GTT​CCA​CCT​CTC​CCA​GCA​TA	CCG​TGA​TAA​TGA​ACC​AGC​GG	
HSPG2	GAG​CCC​ACC​ACA​CTA​CTT​CT	ATC​AGG​TTT​CGG​CAG​GTA​CA	
PROM1	CAA​ACA​GAC​CAA​GGA​TGC​CC	GCT​TGG​TCT​GAT​GCT​ATC​GC	
FGFBP1	ACA​CTC​ACA​GAA​AGG​TGT​CCA	CTG​AGA​ACG​CCT​GAG​TAG​CC	
GAPDH	CCA​CCC​AGA​AGA​CTG​TGG​AT	CAC​ATT​GGG​GGT​AGG​AAC​AC	

### 2.10 Western blot analysis

Cells were seeded at a density of 3 × 10^6^ in 100-mm culture dishes and treated with or without B4 at different concentrations, for 72 h. After the treatment period, the cells were washed (twice) with PBS and then added with 100–120 µL of Radio-immunoprecipitation assay (RIPA) buffer containing Phenylmethanesulfonyl fluoride (PMSF) at a ratio of 50:1 (RIPA: PMSF) for approximately 30 min. A Solarbio (US) BCA assay was performed to quantify the proteins. After adding a 5X loading buffer, the protein extracts were denatured at 95°C for 3 minutes and stored at −20°C until further use. Equal amounts of protein extracts (50 μg) were separated by 10%–15% SDS-PAGE at 100 V and then electro-blotted onto a PVDF membrane (0.22 µM) at 220 mA for approximately 2 hours, based on the molecular weight of the proteins. The blotted proteins were then blocked with 5% BSA for 1 hour at room temperature. Then, the membrane was subjected to an overnight incubation at 4°C with specific primary antibodies. Afterward, the membrane was washed (3 times) with TBS for 15 min each and then incubated with a secondary antibody for 4 hours at room temperature. The membranes were then washed with TBS for 15 min. Following the washing steps, the membrane was treated with an ECL buffer from New Sai Mei Biotechnology Co., Ltd. (CN) and visualized using the Bio-RAD ChemiDoc XRS + imaging system (US) with Image Lab 4.0 software to analyze the band intensities (Varier et al., 2022).

### 2.11 Combinational treatment

To further analyze the effect of B4 in heart H9C2 cells, the cells were treated with an angiotensin-receptor blocker, Candesartan cilexetil, (CC). The cells were exposed to different concentrations of CC (10, 20, 30, 40, and 50 µM) in triplicates. A control group was given 0.01% DMSO. Furthermore, the cells were treated with DMSO, CC (10 μM), CC (10 μM) + B4 (27.6 µM), and CC (10 μM) + B4 (30 µM) for 72 h. The treated cells were also analyzed for gene and protein expression levels of FGFBP1.

### 2.12 Cloning and generation of overexpression plasmid

The cells were transfected with Lipofectamine 3000 (Transfection reagent; Thermo Fisher Scientific-US) and either pEX-2 containing FGFBP1 cDNA (pGCMV/MCS/IRES/EGFP/Neo; Gene ID: NM_005130.5; GenePharma, CN) or an empty vector onto a 6-well plate. To select the transfected cells, they were treated with 1.5 mg/mL neomycin (Sigma Aldrich, United States) for 2 weeks. Following this, the cells were further expanded with 0.7 mg/mL neomycin. The overexpression of FGFBP1 was evaluated using RT-qPCR and Western blot analysis (Hu et al., 2022).

Later, the OCI_LY 10 cells were added with the diluted stock solutions in DMEM and incubated for 24 h at 37°C and 5% CO_2_. Then, the culture medium was changed, and the cells were grown till stable FGFBP1-overexpressed cells were obtained. Consecutively, the FGFBP1-overexpressed cells were treated with various concentrations of B4 (10, 20, 30, 40, and 50 µM) in triplicates for 24, 48, and 72 h. Then, cell viability was assessed using the MTT assay. Additionally, cells treated with or without B4 (27.6 µM) were analyzed for gene and protein expression levels of FGFBP1 (Hu et al., 2022).

### 2.13 Protein-drug binding: Drug affinity responsive target stability (DARTS)

The DARTS assay was performed in OCI_LY10 cells with slight modifications (Thorne et al., 2010; Egnatchik et al., 2017). Initially, approximately 1 × 10^6^ untreated OCI_LY10 cells were lysed on ice for 30 min using a nuclear protein and cytoplasmic extraction reagent (#P0027, Beyotime, CN). After quantifying the protein content using the BCA method, the cell lysate (500 μg total protein, 5 μg/μL) was supplemented with B4 (27.6 µM). The samples were gently mixed to ensure proper binding between the ligand and protein target, followed by incubation for 2 hours at room temperature. Next, aliquots of the lysates with B4 (27.6 µM) were digested using pronase at specific ratios: 1:1000 (+), 1:2000 (++), and 1:3000 (+++) (w/w) for precisely 2 hours. The digested lysates were mixed with loading buffer and boiled at 95°C for 3 minutes. The resulting samples were separated using SDS-PAGE and subjected to Western blot analysis.

### 2.14 Lymphoma C57BL/6 mice model

Animal care and procedures were conducted in accordance with the criteria for the use of laboratory animals. The animals used in the study were purchased from Beijing Huafukang Biotechnology Co., Ltd., CN. The animal protocol complies with ARRIVE guidelines and was approved by the Guizhou Medical University Animal Care Committee, following the guidelines set by the China Council of Animal Care (2302087). The EL4 cells (1 × 10^6^) were intravenously injected into six-week-old C57BL/6 mice (n = 7) to induce lymphoma. Forty-8 hours after this initial injection, the mice were treated every other day for 2 weeks with B4 at a dose of 3 mg/kg body weight (a total of seven treatments). A nonparametric Kaplan-Meier analysis was performed to assess the survival rate of the animals (Gajendran et al., 2020).

### 2.15 Histopathological examination

The lymph node and spleen tissues from vehicle and B4-treated mice were fixed in 4% paraformaldehyde for more than 24 h. After fixation, the tissues were dehydrated and embedded in paraffin. The paraffin blocks were then sectioned at a thickness of 3 μm using a paraffin slicer. Following sectioning, the paraffin sections were deparaffinized and subjected to staining with Hematoxylin and Eosin staining. Then, the tissue sections were dehydrated and sealed. For image acquisition and analysis, the tissue sections were examined using a Digital slide scanner (Pannoramic 250, Jinan DANJIER Electronics Co., ltd.) 3DHISTECH (Syrykh et al., 2020).

### 2.16 Immunofluorescence staining and molecular expression analysis of FGFBP1

The lymph node and spleen sections were washed (3 times) with PBS and fixed in 4% paraformaldehyde for 10 min, following the protocol described by Groff et al., 2019. For paraffin sectioning, the samples were deparaffinized and rehydrated through a gradient of ethanol. Then, they were incubated with H_2_O_2_ (3%) for 15 min to block endogenous peroxidase activity. Antigen retrieval was performed by microwave heating in sodium citrate buffer, followed by cooling to room temperature. After blocking with 5% BSA for 2 h, the sections were incubated overnight (4°C) with a primary antibody against FGFBP1 (diluted 1:500). Subsequently, the cells were incubated with a fluorescent secondary antibody for 2 h at room temperature in darkness. Photomicrographs were captured using a Digital trinocular camera microscopic imaging system (Motic China Group Co., Ltd.; BA400Digital). Further, lymph nodes, including both sides of axillary and subiliac lymph nodes, as well as spleen tissues, were collected from treated and untreated B4 mice (Chen et al., 2019). They were then subjected to RT-qPCR and Western blot analysis.

### 2.17 Lymphoma primary cell isolation from patient’s tissue

Patient tissue from (>67-year-old) small intestine lymphoma was obtained from the Affiliated Hospital of Guizhou Medical University. The study involving the use of patient tissue was conducted under the Declaration of Helsinki and the ethical committee of Guizhou Medical University in China (2023/26). Following ethical guidelines, informed consent from the patients was obtained and documented by the ethical committee before the use of the patient’s tissue in the present study.

The collected patient’s tissue was placed in sterile 1X phosphate-buffered saline (PBS) to maintain its integrity and prevent contamination. Subsequently, the tissue was homogenized using an electric homogenization unit to fragment the tissue. The homogenized tissue was washed twice with 1X PBS to remove debris or unwanted substances and passed through a sterile filter with a pore size of 0.22 μm. The filtered cells were then plated in αMEM (alpha-minimum Essential Medium) supplemented with fetal bovine serum (FBS) at a concentration of 15%. To support their growth, the culture medium was further enhanced with 1 U/mL of EPO, 10 ng/mL of stem cell factor (SCF), 100 U/mL of penicillin/streptomycin, and 4 mol/L of 2-mercaptoethanol. The cells were allowed to expand for 2 days until suspension cells were easily discernible (Shaked et al., 2005). Furthermore, the cells were used for downstream applications such as cell viability, RT-qPCR, and Western blot analysis.

### 2.18 Statistical analysis

Unless otherwise specified, all statistical analyses were performed using the ANOVA test and *post hoc* analysis using GraphPad Prism 9 Software (San Diego, CA, United States). The experiments were conducted in triplicate and repeated independently three times to ensure reproducibility. The data obtained from the experiments were expressed as mean ± standard deviation (SD). The significance levels were denoted using the following standard scheme: * (*p* < 0.05), ** (*p* < 0.01), *** (*p* < 0.001), and **** (*p* < 0.0001).

## 3 Results

### 3.1 Anti-proliferative efficacy of B4 leading to apoptosis

**FIGURE 1:**
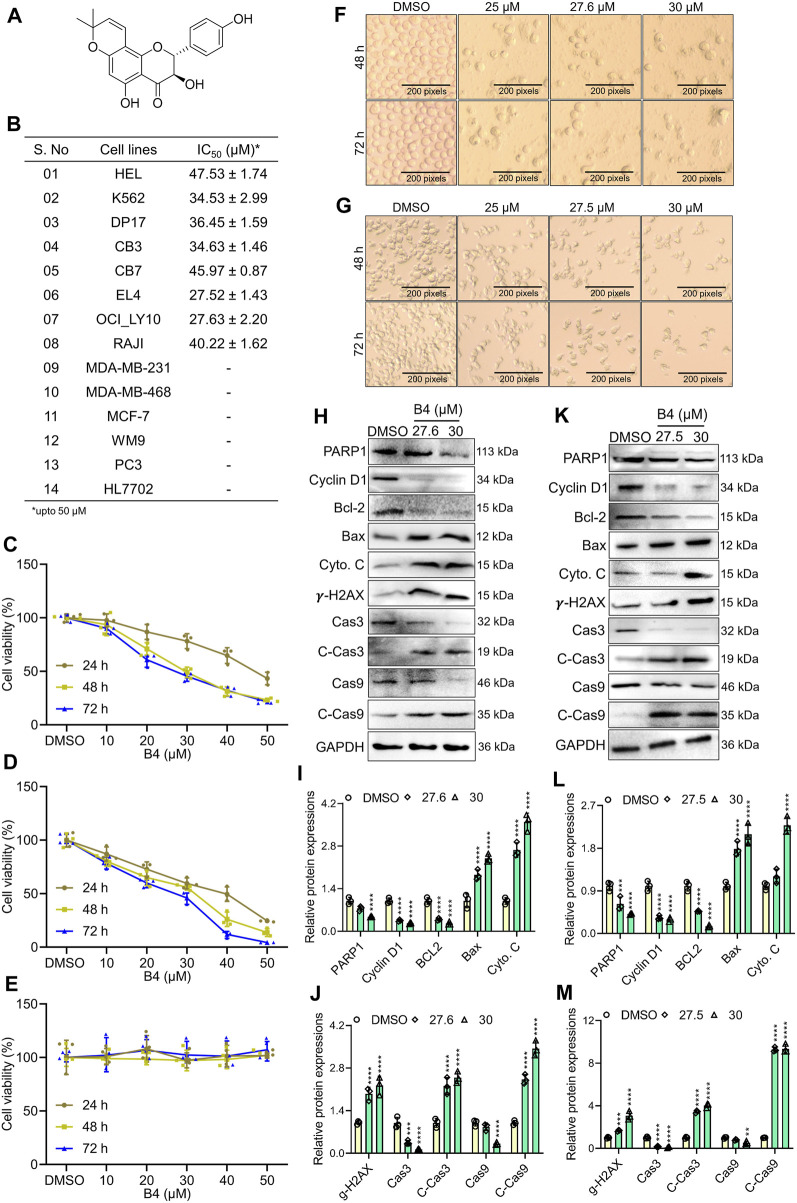
Cytotoxic effects of B4 on the proliferation of cancer cells. **(A)** Chemical structure of B4. **(B)** IC_50_ values of various cancer cell lines after 72 h of incubation with B4 at concentrations up to 50 µM. **(C–E)** Time and dose-dependent viability of OCI_LY10 **(C)**, EL4 **(D)**, and HL7707 **(E)** cells treated with B4. **(F,G)** Morphological changes were observed in OCI_LY10 **(F)** and EL4 **(G)** cells at indicated hours of B4 treatment (100x). **(H–M)** The indicated protein expressions and densitometric analysis of B4-treated OCI_LY10 **(H–J)** and EL4 **(K–M)** cells at 72 h of incubation. The protein expressions are normalized with GAPDH. Data were represented as mean ± SD (*n* = 3); **p < 0.01, ***p < 0.001, ****p < 0.0001, *versus* DMSO.

The B4 (structure shown in Figure 1A) showed a significant therapeutic inhibitory effect on erythro-lymphoblastic cell lines. Compared to melanoma, prostate, and breast cancer cells, the B4 showed better inhibitory action with a lesser inhibitory concentration in mouse (EL4; 27.52 ± 1.43 µM) and human lymphoma (OCI_LY10; 27.63 ± 2.20 µM) cells. Furthermore, B4 could significantly induce dose- and time-dependent inhibition in the growth of OCI_LY10 and EL4 cells (Figures 1B–D; Supplementary Figure S1). However, the non-tumorous HL7707 cells had an insignificant inhibitory effect both dose- and time-dependently (Figure 1E). Additionally, B4 treatment revealed dose-dependent morphological changes in OCI_LY10 and EL4 cells (Figures 1F, G), causing cellular damage through cell-membrane distortions and increased desiccated cells as the B4 concentration increased. To confirm that the cellular loss is triggered by the apoptotic machinery, a Western blot of apoptotic and cell cycle proteins was performed. It revealed a significant decrease in PARP-1, Cyclin D1, Bcl-2, Caspase-3, and Caspase-9 as the B4 concentration increased. Similarly, there was an increase in the protein expression of Cleaved-caspase3, Cleaved-caspase-9, and cytoplasmic Cyto. C, Bax, and gamma-H2AX, dose-dependently in both OCI_LY10 and EL4 cells (Figures 1H–M). These results indicate that B4 can selectively inhibit the growth of lymphoma cells, leading to apoptosis in a time- and dose-dependent manner.

### 3.2 Effect of B4 on lymphoma cells causing intrinsic apoptosis by arresting G1/S cell cycle transition phase

**FIGURE 2:**
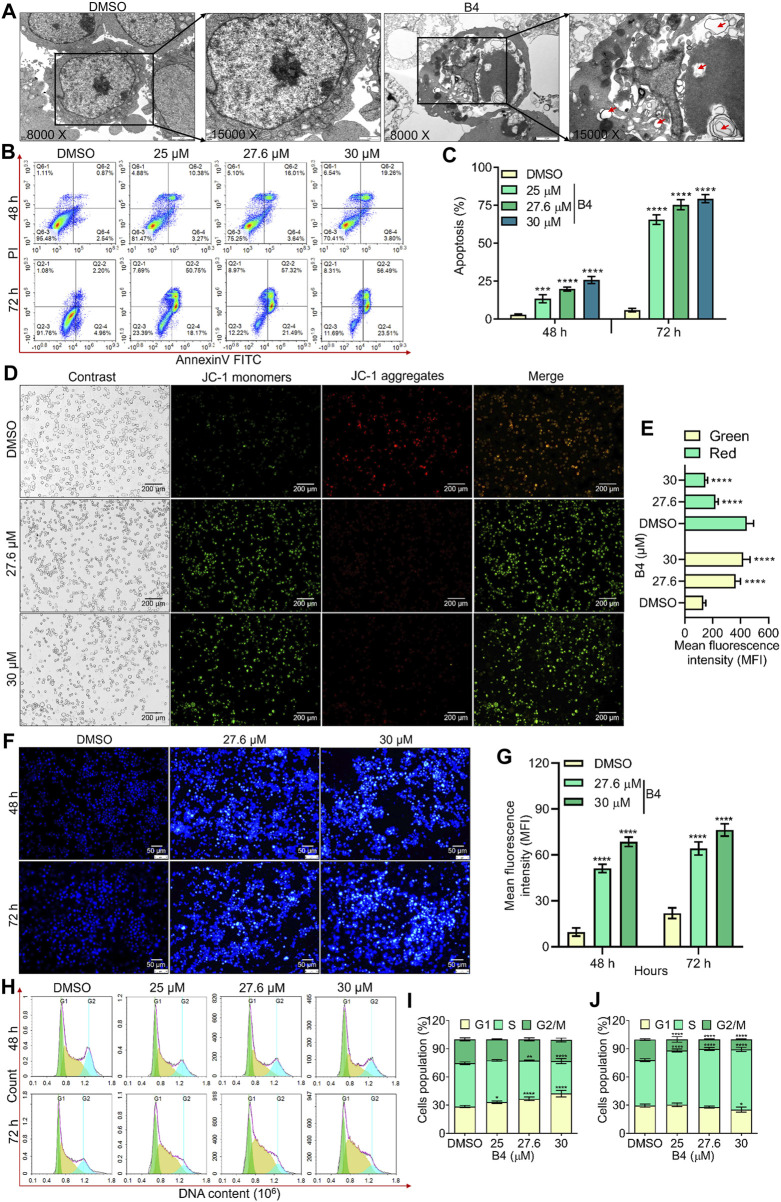
Effect of B4 on apoptosis and cell cycle arrest of OCI_LY10 cells. **(A)** TEM images revealed plasma membrane and mitochondrial damage in cells treated with 27.6 µM of B4 for 72 h (8000x [2 µm] and 15000x [1 µm]). **(B)** Flow cytometry analysis of B4-induced apoptosis in cells at 48 and 72 h. **(C)** Percentage of apoptotic cells treated with B4. **(D)** Mitochondrial damage in cells treated with B4 for 72 h using JC-1 staining (20x). **(E)** Fluorochrome intensity for JC-1 monomers (green) and aggregates (red). **(F)** Fluorescent staining of apoptotic bodies in cells treated with B4 at indicated doses and time points using Hoechst staining (20x). **(G)** Fluorochrome density in apoptotic bodies. **(H)** Cell cycle alterations in B4-treated cells at 48 and 72 h **(I,J)** Densitometry analysis of the cell cycle phases in cells after 48 **(I)** and 72 h **(J)** of B4 treatment. Data were represented as mean ± SD (*n* = 3); *p < 0.05, **p < 0.01, ***p < 0.001, ****p < 0.0001, *versus* DMSO.

Further, TEM analysis was performed on OCI_LY10 cells treated with or without B4 (27.6 µM), confirming that apoptosis is triggered by a mitochondrion-dependent pathway. It revealed damaged mitochondria and autophagosomes containing dense organelles, with a shredded plasma membrane, in B4-exposed cells when intact mitochondria and plasma membrane were observed in DMSO-exposed cells (Figure 2A). Breakage of the nuclear membrane was also observed in B4-treated cells, causing the invasion of autophagosomes and damage to the nucleus. However, in DMSO-treated cells, the nucleus was intact with a distinct nuclear membrane. The apoptotic-mediated cellular loss was further analyzed using annexinV-FITC-stained flow cytometry. This revealed a dose- and time-dependent progression of apoptosis, up to 75% and 26%, respectively, in B4-treated OCI_LY10 and EL4 cells compared to DMSO-treated cells after 72 h of incubation at a concentration of 30 µM (Figures 2B, C; Supplementary Figure S2A, B). JC-1 staining showed significant dose-dependent mitochondrial damage in cells post-B4 treatment, leading to the formation of JC-1 monomers instead of aggregates (Figures 2D, E; Supplementary Figures S2C, D). There was a higher formation of apoptotic bodies in B4-treated cells in both a dose- and time-dependent manner (Figures 2F, G; Supplementary Figures S2E, F), suggesting activation of the cell death receptor compared to DMSO-treated cells. PI-stained cell cycle analysis revealed that B4-treated cells are arrested at the G_1_/S transition stage compared to DMSO-treated cells, dose-dependently, at both 48 and 72 h of incubation in OCI_LY10 (Figures 2H–J) and EL4 cells (Supplementary Figures S2G–I). These results indicate that B4 can selectively inhibit the growth of lymphoma cells by triggering apoptosis and causing cell cycle arrest at the G_1_/S transition stage.

### 3.3 B4 extends the survival rates of lymphoma mice

**FIGURE 3:**
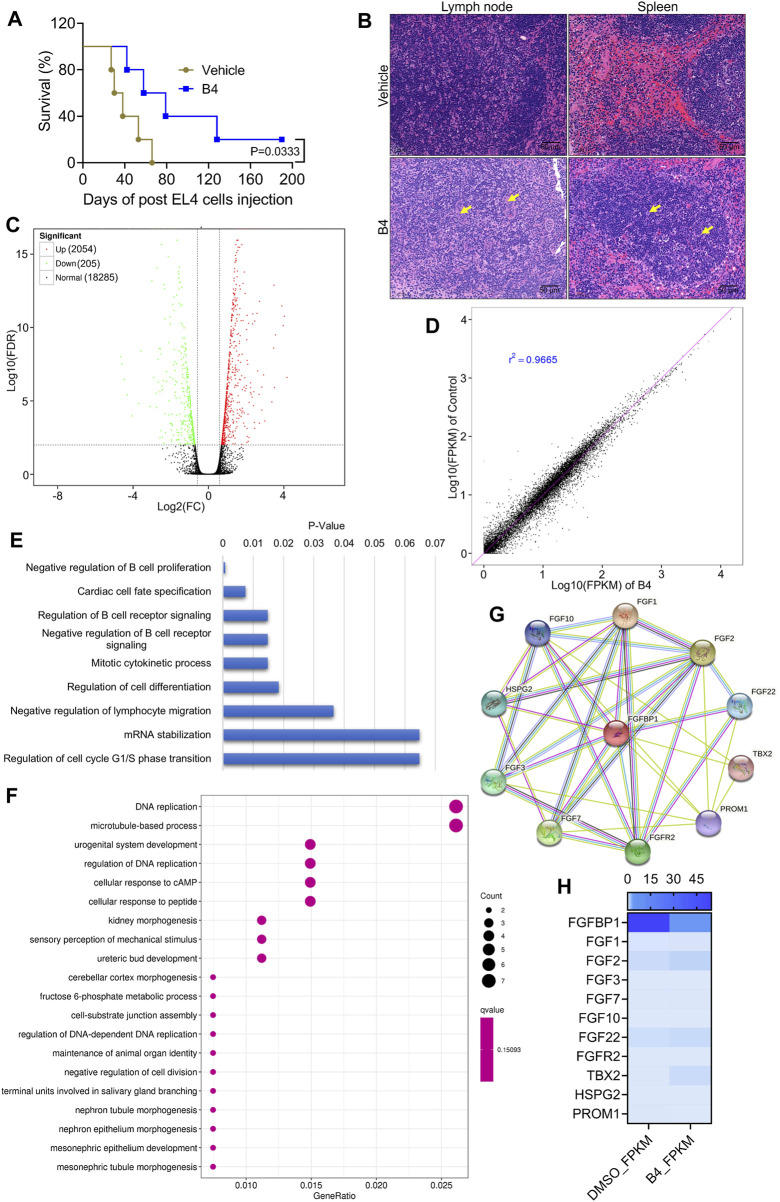
Molecular intervention of B4 in lymphoma cells to increase mice survival rates. **(A)** The survival rate of EL4-cells injected mice treated with B4 (3 mg/kg; n = 7). **(B)** H&E staining of lymph node and spleen tissues from vehicle and B4-treated mice. **(C,D)** Volcano **(C)** and co-relation mapping **(D)** of EL4 cells treated with B4 (26.5 µM) for 72 h **(E,F)** KEGG pathway enrichment **(E)** and molecular function **(F)** plots of differentially expressed genes, in OCI_LY10 cells after 72 h of B4 treatment. **(G,H)** STRING database analysis **(G)** and heatmap **(H)** of B4-induced FGFBP1-related molecules identified through RNA sequencing.

When EL4 cells were injected into C57BL/6 mice and treated with 3 mg/kg B.W of B4, there was a significant (*p* = 0.0333) increase in the survival rate of mice, up to 187 days, compared to the DMSO-treated mice, which expired by 66 days after cell injection (Figure 3A). Moreover, H&E staining of lymph node and spleen in control mice showed increased follicular infiltration of cells and larger accumulation of collagen, compared to B4-treated mice, which showed nearly normal functioning with densely packed lymphocytes in the lymph node and spleen (Figure 3B). These observations suggest that B4 could extend the mice survival rates.

### 3.4 B4 inhibits FGFBP1, leading to cell death in lymphoma cells

**FIGURE 4:**
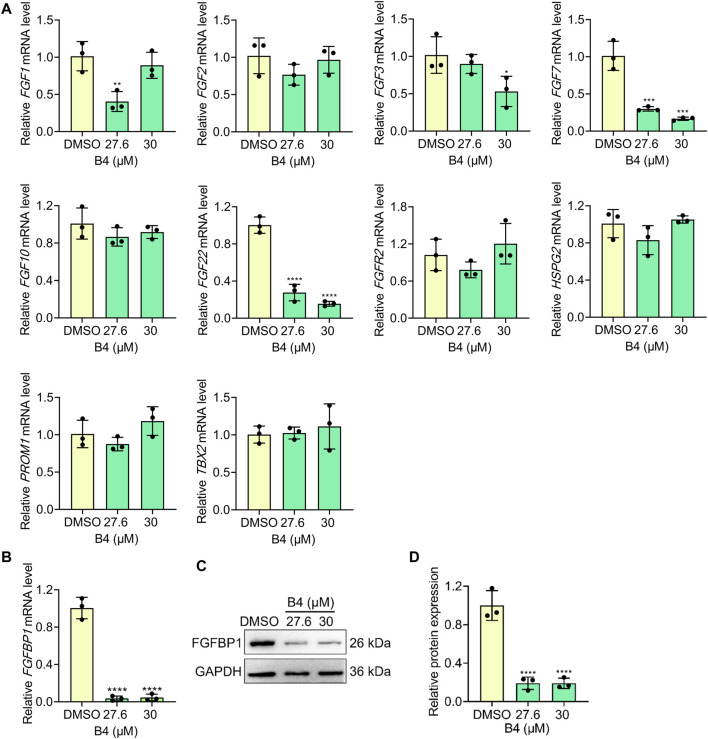
Effect of B4 on FGBP1- related molecules in OCI_LY10 cells. **(A,B)** RT-qPCR analysis of indicated genes in cells treated with different concentrations of B4 at 72 h **(C,D)** FGFBP1 protein expressions **(C)** and densitometry **(D)** of cells treated with B4 for 72 h. Data were represented as mean ± SD (*n* = 3); *p < 0.05, **p < 0.01, ***p < 0.001, ****p < 0.0001, *versus* DMSO.

The molecular intervention of B4 in EL4 cells was elucidated using RNA-Seq analysis. The results indicated that out of 20,544 genes, B4 treatment significantly upregulated 2,054 genes and downregulated 205 genes compared to DMSO-treated cells (Figures 3C, D). Furthermore, biological process and pathway enrichment analysis of the downregulated genes revealed that the significantly altered genes are involved in the negative regulation of B-cell proliferation (*p* = 0.0008) and DNA replication (*p* = 0.0005), followed by the cardiac cell fate specification (*p* = 0.007) pathway genes (Figures 3E, F). These observations suggest that differentially expressed genes could enhance cell death. Based on the enrichment analysis of downregulated genes, the significantly altered gene was found to be FGFBP1. Further analysis of its associated FGF signaling genes showed insignificant variations in their genetic expressions in B4-treated cells compared to DMSO-treated cells (Figures 3G, H). In OCI_LY10 cells, there was a reduction in gene expressions of FGF1, FGF3, FGF7, and FGF22, while FGF2, FGF10, FGFR2, HSPG2, PROM1, and TBX2 levels remained unchanged (Figure 4A). However, in EL4 cells, there was a reduction in gene expressions of FGF2, FGF7, FGF10, and TBX2, while FGFR2, HSPG2, PROM1, FGF1, FGF3, and FGF22 levels remained normal (Supplementary Figure S3A). Notably, there was a consistent drastic reduction in the FGFBP1 gene level in both OCI_LY10 and EL4 cells, dose-dependently, upon B4 treatment (Figure 4B; Supplementary Figure S3B). A similar reduction of protein levels was seen in OCI_LY10 (Figures 4C, D) and EL4 (Supplementary Figures S3C, D) cells. Nevertheless, no changes were detected in the expression of the FGFBP1 gene and protein levels in leukemic (HEL) and breast cancer (MDA-MB-468) cells (Supplementary Figure S4), suggesting that B4 specifically acts on lymphoma, unlike in other cancer cells.

### 3.5 B4 treatment in combination with CC enhances the anti-lymphoma effects

Patients with hypertension often exhibit high levels of FGFBP1 as a pathology marker [23]. Interestingly, when B4 was administered to mouse heart myoblast cells (H9C2 cells), there was a dose-dependent increase in the expression of FGFBP1 at the gene level (Supplementary Figure S5A) and protein levels (Supplementary Figures S5B, C). However, when a combination of B4 was administered with CC, a hypertension treatment drug, there was a reversal in the expression of FGFBP1 (Supplementary Figures S5D–F). H&E staining revealed no significant alterations in the heart tissues of the vehicle and B4-treated lymphoma mice (Supplementary Figure S5G). The same was observed in the expression of FGFBP1-positive cells in the heart tissues of the vehicle and B4-treated mice (Supplementary Figure S5H). Similarly, no changes were observed in the gene and protein levels of FGFBP1 in the heart tissues of the vehicle and B4-treated mice (Supplementary Figures S5I–K). These results suggest that B4 specifically alters FGFBP1 in lymphoma and heart cells. Consequently, combining these treatments could potentially provide a novel approach to treating lymphoma in individuals with hypertension.

### 3.6 Affinity binding of B4 with FGFBP1 induces cell death

**FIGURE 5:**
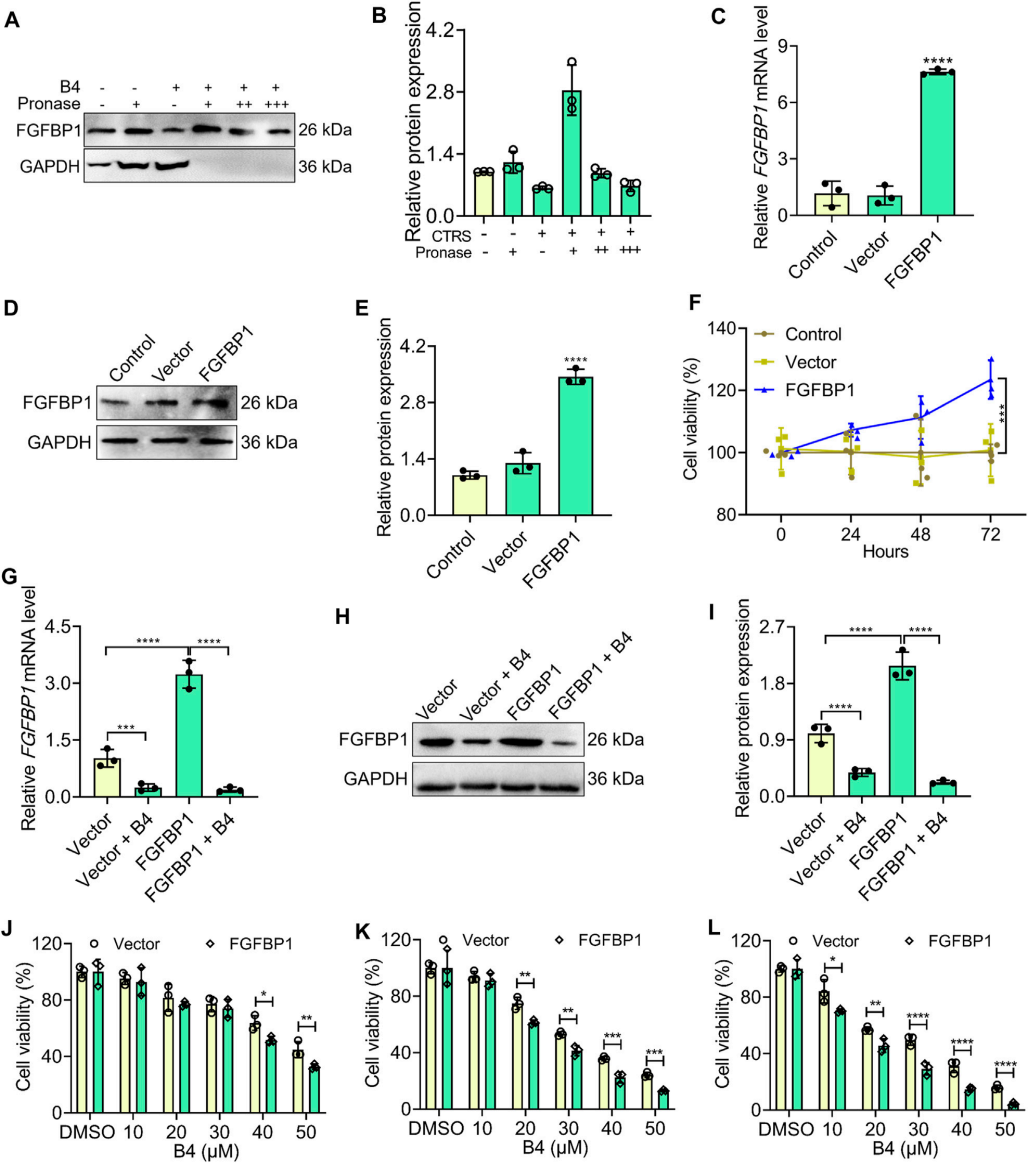
Affinity binding analysis of B4 with FGFBP1 in OCI_LY 10 cells. **(A,B)** Validation of B4 affinity binding interaction with FGFBP1 proteins using the DARTS assay. Western blot **(A)** and graph **(B)** show the levels of FGFBP1, and GAPDH (loading control) in response to varying concentrations of pronase (+: 1:1000; ++: 1:2000; +++: 1:3000) and B4 (27.6 µM) in OCI_LY10 cells. **(C)** RT-qPCR analysis of FGFBP1 in FGFBP1-overexpressed OCI_LY10 cells. **(D,E)** Protein expression **(D)** and densitometry analysis **(E)** of FGFBP1 in FGFBP1-overexpressed OCI_LY10 cells. **(F)** Cell viability of control, vector and FGFBP1-overexpressed OCI_LY10 cells. **(G)** RT-qPCR analysis of FGFBP1 in FGFBP1-overexpressed OCI_LY10 cells treated with B4. **(H,I)** Protein expression **(H)** and densitometry analysis **(I)** of FGFBP1 in FGFBP1-overexpressed OCI_LY10 cells treated with B4. **(J–L)** Inhibition of cell growth by different concentrations of B4 in vector and FGFBP1-overexpressed OCI_LY10 cells at 24 **(J)**, 48 **(K)**, and 72 h **(L)**. Data were represented as mean ± SD (*n* = 3); *p < 0.05, **p < 0.01, ***p < 0.001, ****p < 0.0001, *versus* as indicated in figures.

The targeted therapeutic efficacy of B4 was assessed through DARTS and overexpression plasmid studies. DARTS analysis demonstrated that B4 could strongly bind to FGFBP1 at a dosage of 27.6 µM in OCI_LY10 cells (Figures 5A, B). Interestingly, when FGFBP1 was overexpressed (Figures 5C–E), OCI_LY10 cells exhibited a significant increase in cell proliferation compared to vector cells (Figure 5F). After treatment with B4, the FGFBP1-overexpressed cells showed lower mRNA downregulation when compared to vector + B4 treated cells (Figure 5G). However, when B4 was administered to FGFBP1-overexpressed cells, it was more effective in inhibiting FGFBP1 protein levels compared to vector + B4 treated cells (Figures 5H, I). Additionally, the overexpressed cells were more susceptible to B4 treatment than the vector cells, indicating that B4 is a potential ligand for FGFBP1 (Figures 5J–L). These results confirm that B4 can bind strongly to FGFBP1, leading to its activation and subsequent cellular loss.

### 3.7 FGFBP1 induces lymphoma suppression in mice and patient-derived primary cells

**FIGURE 6:**
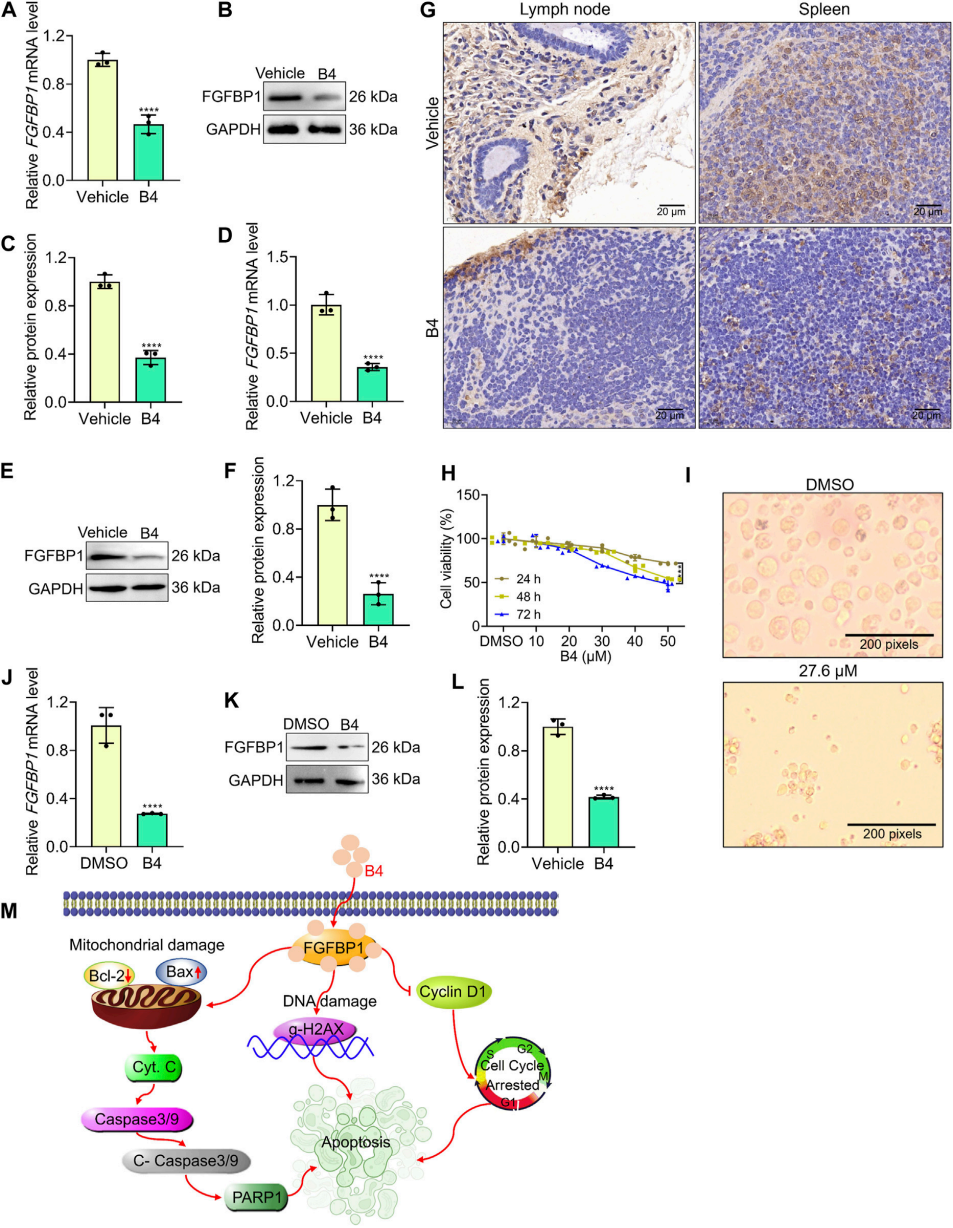
B4 initiates lymphoma inhibition in pre-clinical models. **(A–F)** RT-qPCR and Western blot analysis with densitometry of FGFBP1 in lymph node **(A–C)** and spleen **(D–F)** tissues from vehicle and B4-treated mice. **(G)** Immunofluorescence of lymph node and spleen tissues from vehicle and B4-treated mice against FGFBP1. **(H)** Time and dose-dependent inhibitory effect of B4 in primary lymphoma cells. **(I)** Morphological variations in primary lymphoma cells treated with B4 (27.6 µM) at 72 h **(J)** RT-qPCR analysis of FGFBP1 in primary lymphoma cells treated with B4 at 72 h **(K,L)** Protein expression **(K)** and densitometry analysis **(L)** of FGFBP1 proteins in primary lymphoma cells treated with B4 at 72 h. **(M)** The proposed molecular therapeutic mechanism of B4 in lymphoma preclinical models. Data were represented as mean ± SD (*n* = 3); ****p < 0.0001, *versus* as indicated in figures.

The lymph node and spleen tissues of mice administered with B4 showed a significant decrease in gene and protein levels of FGFBP1 (Figures 6A–F) compared to the vehicle group. Additionally, the number of FGFBP1-positive cells in the lymph node and spleen of B4-treated mice was significantly lower than in the vehicle group (Figure 6G). These results indicate that B4 potentially enhances FGFBP1 inhibition, thereby improving the survival rates of lymphoma mice (Figure 3A).

Furthermore, when primary cells isolated from lymphoma patients were treated with B4, there was a dose-dependent decrease in cell viability (Figure 6H). Notably, the cells exhibited significant morphological deformations and cellular loss after B4 treatment (Figure 6I). Moreover, the gene and protein levels of FGFBP1 were significantly reduced in the primary cells following B4 treatment (Figures 6J–L). These findings provide further evidence of the therapeutic efficacy of B4 against lymphoma, achieved through the FGFBP1-mediated suppression of lymphoma.

## 4 Discussion

In recent times, the number of lymphoma cases and deaths worldwide has highly increased. The use of phytochemicals in regulating B-lymphocyte expansion has shown promising results in recent studies (Liu et al., 2021; Qiao et al., 2022). This study presents such a novel mechanism by which B4 inhibits the development of lymphoma by targeting FGFBP1-mediated signaling, leading to caspase-dependent intrinsic apoptosis both *in vitro* and *in vivo*. These conclusions offer an appreciated understanding of the potential usage of B4 for lymphoma treatment and its therapeutic application.

Regulatory dysregulation of the cell death processes plays a central role in cancer development, where the normal mechanisms of cell cycle rheostat become diminished, leading to excessive cell proliferation and abridged cell removal (Zhou et al., 2023). Apoptotic suppression is a dominant factor in the progression of certain cancers (Ma et al., 2023). Tumorous B-cells can acquire resistance to apoptosis through the expression of anti-apoptotic proteins like Bcl-2 or the mutation of pro-apoptotic proteins like Bax (Zhang et al., 2023). Notably, some types of B-cell lymphoma show overexpression of Bcl-2, providing solid evidence that cell death dysregulation contributes to cancer development (Zhu et al., 2022). Hence, incapacitating mechanisms that hamper apoptosis can have significant insinuations in cancer therapy. Apoptosis can ascend through either the intrinsic pathway, which is mitochondria-dependent, or the extrinsic pathway, which is death receptor-dependent. The intrinsic pathways comprise the initiation of Caspase-3, leading to DNA fragmentation, deprivation of cellular and nuclear proteins, establishment of apoptotic bodies, protein cross-linking, expression of ligands for phagocytic cell receptors, and subsequent uptake by phagocytic cells. According to Ma et al., 2023, the extrinsic pathway not only activates the caspase-dependent cell death pathway but also triggers a parallel, caspase-independent cell death pathway through the induction of single-stranded DNA impairment. In our study, we found a significant drop in the propagation of lymphoma cells following treatment with B4. This effect was mediated through the intrinsic apoptosis pathway, as evidenced by the increased expression of pro-apoptotic proteins such as Bax, Cyto. C, Cleaved-caspase 3, Cleaved-caspase 9, and gamma-H2AX. Conversely, we observed a decrease in the expression of anti-apoptotic proteins like Bcl-2, Caspase 3, Caspase 9, PARP-1, and the cell cycle G_1_/S transition phase marker protein, Cyclin D1. These findings were further supported by the development of apoptotic bodies and noticeable adjustments in the cell cycle observed in lymphoma cells treated with B4. Importantly, our study also demonstrated the potential therapeutic benefits of B4 in lymphoma treatment, as it significantly increased the survival rates of a lymphoma mouse model and patient-derived primary lymphoma cells. These results highlight the promising role of B4 in improving outcomes for lymphoma patients.

In cancer therapeutics by small molecules, the induction of apoptosis through molecular intervention needs an initiator molecule that triggers programmed cell death. In our study, we utilized RNA sequencing to identify the target-specific initiator molecules tangled in the apoptotic response of lymphoma cells to B4 treatment. We found that B4 can trigger apoptosis by FGFBP1 inhibition in lymphoma cells. The FGFBP family consists of chaperone proteins that bind to fibroblast growth factors (FGFs) and enhance their biological activity (Tassi and Wellstein, 2006). FGFBP1, in particular, has been shown to bind to multiple FGFs and facilitate their transport from the extracellular matrix to target cells expressing FGF receptors (Tassi et al., 2011). This finding aligns with previous research demonstrating that FGFBP1 knockdown exerts tumor-inhibiting effects in colon carcinoma, including anti-proliferative and pro-apoptotic effects leading to cell cycle arrest (Schulze et al., 2011). The role of FGFBP1 in promoting cell proliferation and exerting anti-apoptotic effects has been established in connection with FGF signaling (Wang F. et al., 2021; Qiu et al., 2023; Zhang et al., 2023). Interestingly, our study revealed a cell-specific reduction in FGFBP1 levels in lymphoma cells upon B4 treatment, with negligible effects on FGF signaling molecules like FGF1, FGF2, FGF3, FGF7, FGF10, FGF22, FGFR2, HSPG2, PROM1, and TBX21. Even though some of the FGF-signaling molecules were differentially expressed after B4 treatment, unlike FGFBP1, their effects were inconsistent in both OCI_LY 10 and EL4 cells. Also, we utilized the DARTS assay to validate the affinity binding of FGFBP1 by B4 in OCI_LY10 cells, thereby offering supplementary evidence for the role of FGFBP1 in the apoptotic response to B4 treatment. These variations in the reduction of FGFBP1 mRNA levels could probably be due to a feedback mechanism of FGFBP1 mediated regulatory response, which requires a deeper understanding in the future. Then, our findings demonstrated that cells overexpressing FGFBP1 exhibited amplified sensitivity to B4 treatment, resulting in enhanced cell inhibition effects compared to vector cells. This mechanism of action of FGFBP1 inhibition by B4 was further confirmed in patient-derived primary lymphoma cells and mouse models, providing additional support for its pharmacotherapeutic significance. Therefore, this study emphasizes the potential significance of FGFBP1 as an originator of caspase-dependent apoptosis in lymphoma cells.

The literature has indeed suggested an amplified expression of FGFBP1 in the heart cells of hypertensive patients (Tomaszewski et al., 2011; Tassi et al., 2018). On the other hand, Candesartan cilexetil, an angiotensin-receptor blocker (ARB), is frequently used to treat hypertension (Ni et al., 2020; Xu et al., 2021). Consistent with these findings, our research also demonstrated that treatment with B4 occasioned an increased level of FGFBP1 levels in heart H9C2 cells, hypothetically contributing to hypertension. However, this effect was effectively thwarted by the co-administration of Candesartan cilexetil with B4, suggesting a probable substitute remedy for hypertensive lymphoma patients. This combined treatment approach led to a reduction in FGFBP1 levels, offering a potential strategy for managing hypertension in the context of lymphoma treatment.

## 5 Conclusion

In conclusion, the research findings indicate that the B4 treatment in lymphoma cells results in a durable binding to FGFBP1. This binding affinity triggers the initiation of Caspase 3 and 9, leading to the cleavage of mitochondria and the subsequent release of Cyto. c into the cytosol. Consequently, the expression of Bax is increased while the expression of Bcl-2 and PARP-1 is decreased, causing DNA damage through the activation of gamma-H2AX expression. Furthermore, the treatment with B4 inhibits the proliferation of lymphoma cells by interfering with the G_1_/S transition phase of the cell cycle, achieved by reducing Cyclin D1 levels (Figure 6M. These findings suggest that targeting specific FGFBP1-inhibition by a pharmacological intervention could be a beneficial strategy for dealing with lymphoma.

## Data Availability

The original contributions presented in the study are publicly available. This data can be found here: https://www.ncbi.nlm.nih.gov/bioproject/PRJNA1118420/
