# Supplementary material for: B4 suppresses lymphoma progression by inhibiting fibroblast growth factor binding protein 1 through intrinsic apoptosis
Source: Front Pharmacol. 2024 Jun 28;15:1408389. doi: 10.3389/fphar.2024.1408389 (PMC11239434; doi:10.3389/fphar.2024.1408389)
Supplement: Supplementary file 1 [file DataSheet1.docx]

Supplementary Material

#
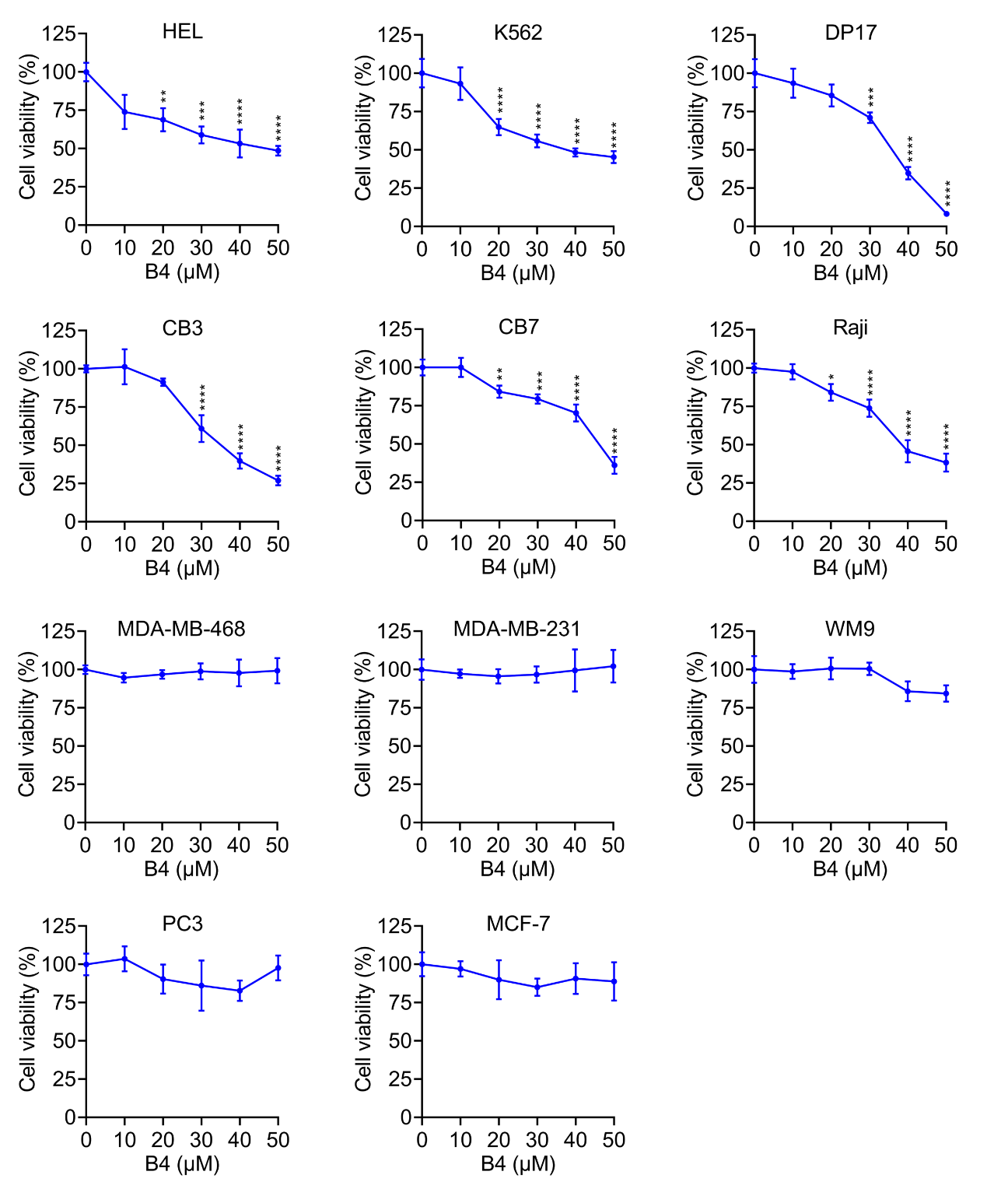
Supplementary Figures

**Supplementary Figure 1** B4 selectively inhibits cell proliferation in the indicated cancer cell lines at 72 hours, tested upto 50 µM. Data were represented as mean ± SD (n = 3); **p < 0.01, ***p < 0.001, ****p < 0.0001, versus DMSO.


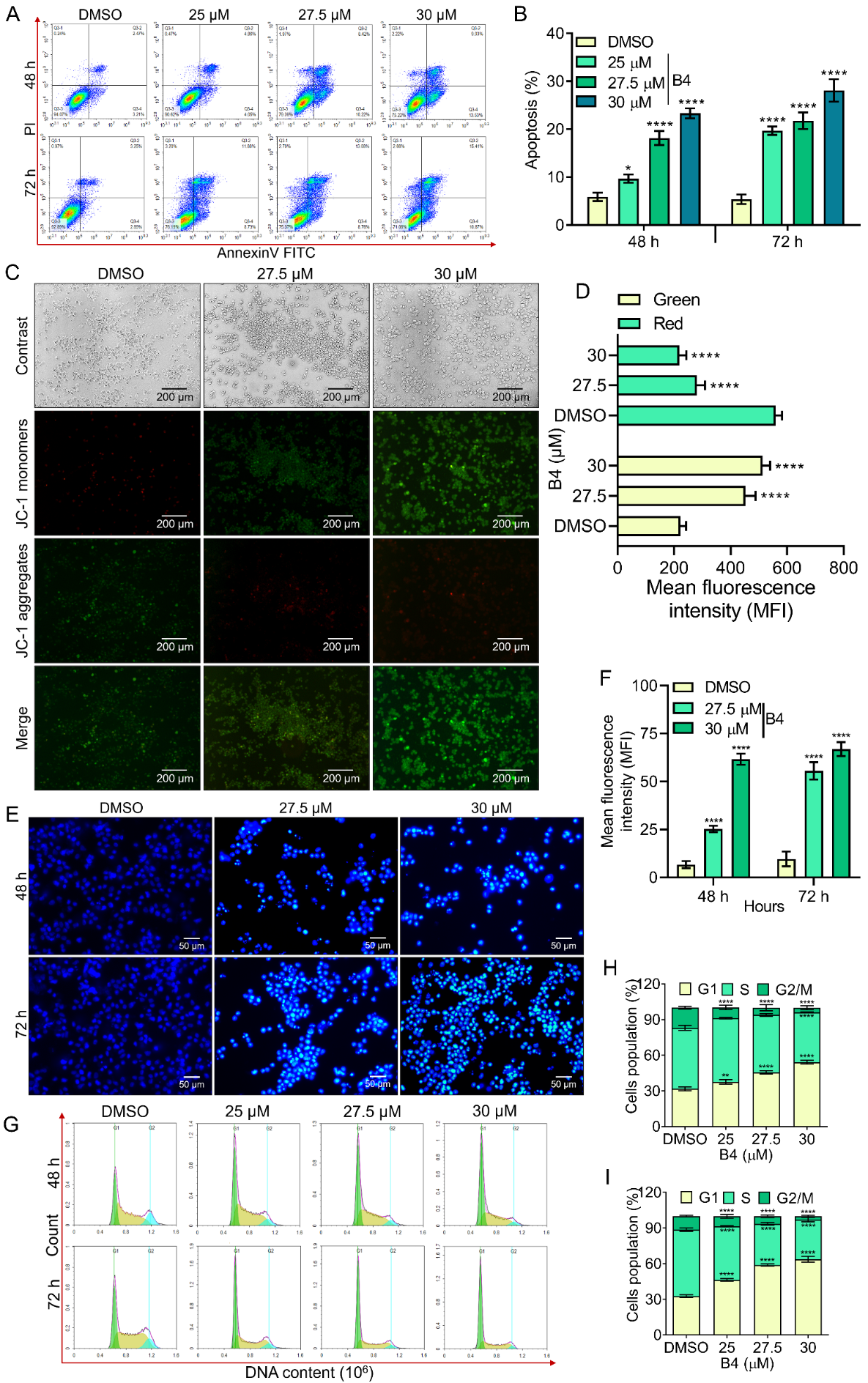


**Supplementary Figure 2** Effect of B4 on apoptosis and cell cycle arrest of EL4 cells. (A) Flow cytometry analysis of B4-induced apoptosis in cells at 48 and 72 hours. (B) Apoptotic percentage of EL4 cells treated with B4. (C) Mitochondrial damage in EL4 cells treated with B4 for 72 hours using JC-1 staining. (D) Fluorochrome intensity for JC-1 monomers (green) and aggregates (red). (E) Fluorescent staining of apoptotic bodies in EL4 cells treated with B4 at indicated doses and time points using Hoechst staining. (F) Fluorochrome density in apoptotic bodies. (G) Cell cycle alterations in B4-treated EL4 cells. (H, I) Densitometry analysis of the cell cycle phases in cells at 48 (H) and 72 hours (I) of B4 treatment. Data were represented as mean ± SD (n = 3); *p < 0.05, **p < 0.01, ****p <
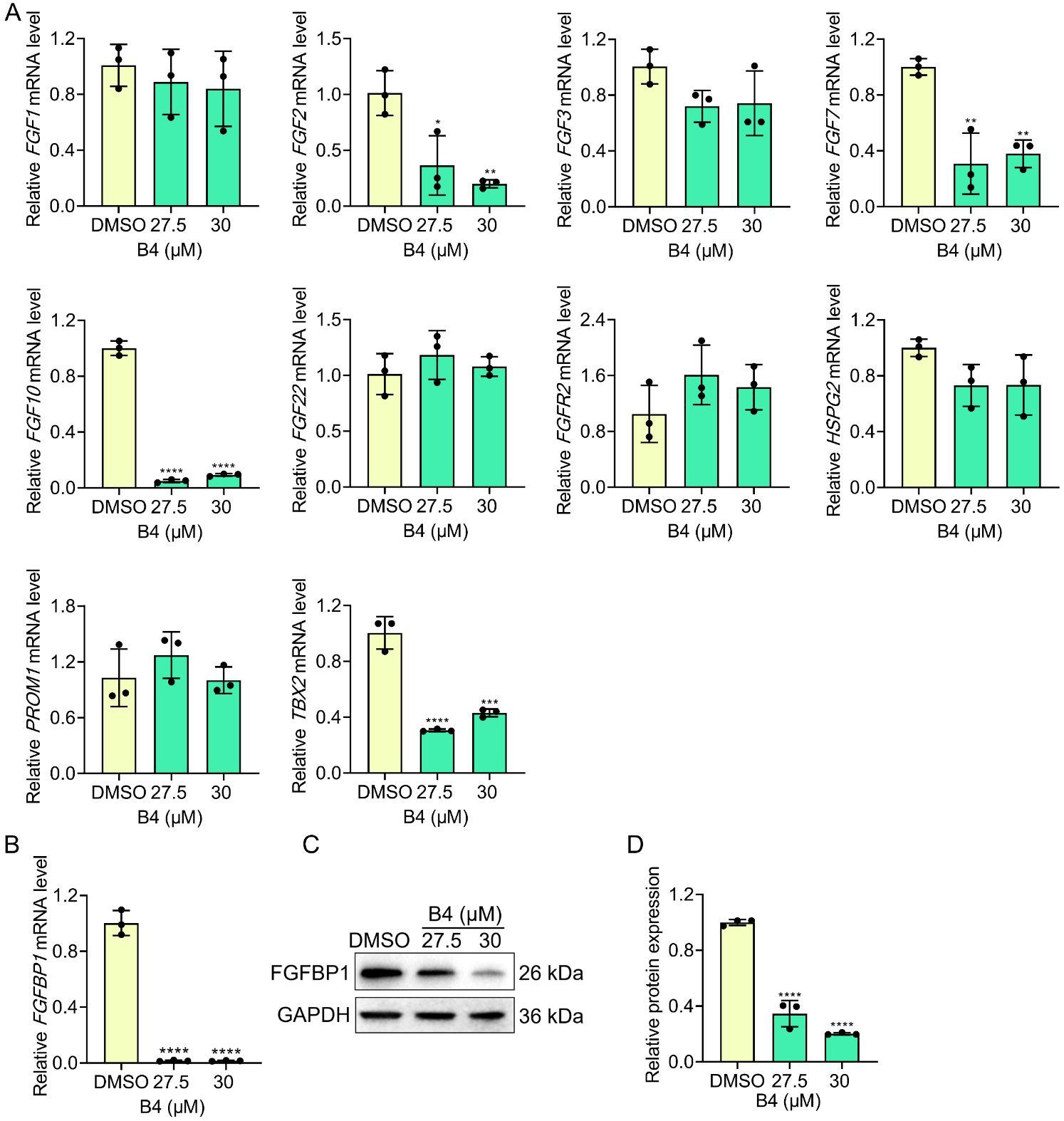
0.0001, versus DMSO.

**Supplementary Figure 3** Effect of B4 on FGBP1-related molecules in EL4 cells. (A, B) RT-qPCR analysis of indicated genes in cells treated with different concentrations of B4 at 72 hours. (C-D) FGFBP1 protein expressions (C) and densitometry (D) of cells treated with B4 for 72 hours. Data were represented as mean ± SD (n = 3); *p < 0.05, **p < 0.01, ***p < 0.001, ****p < 0.0001, versus DMSO.


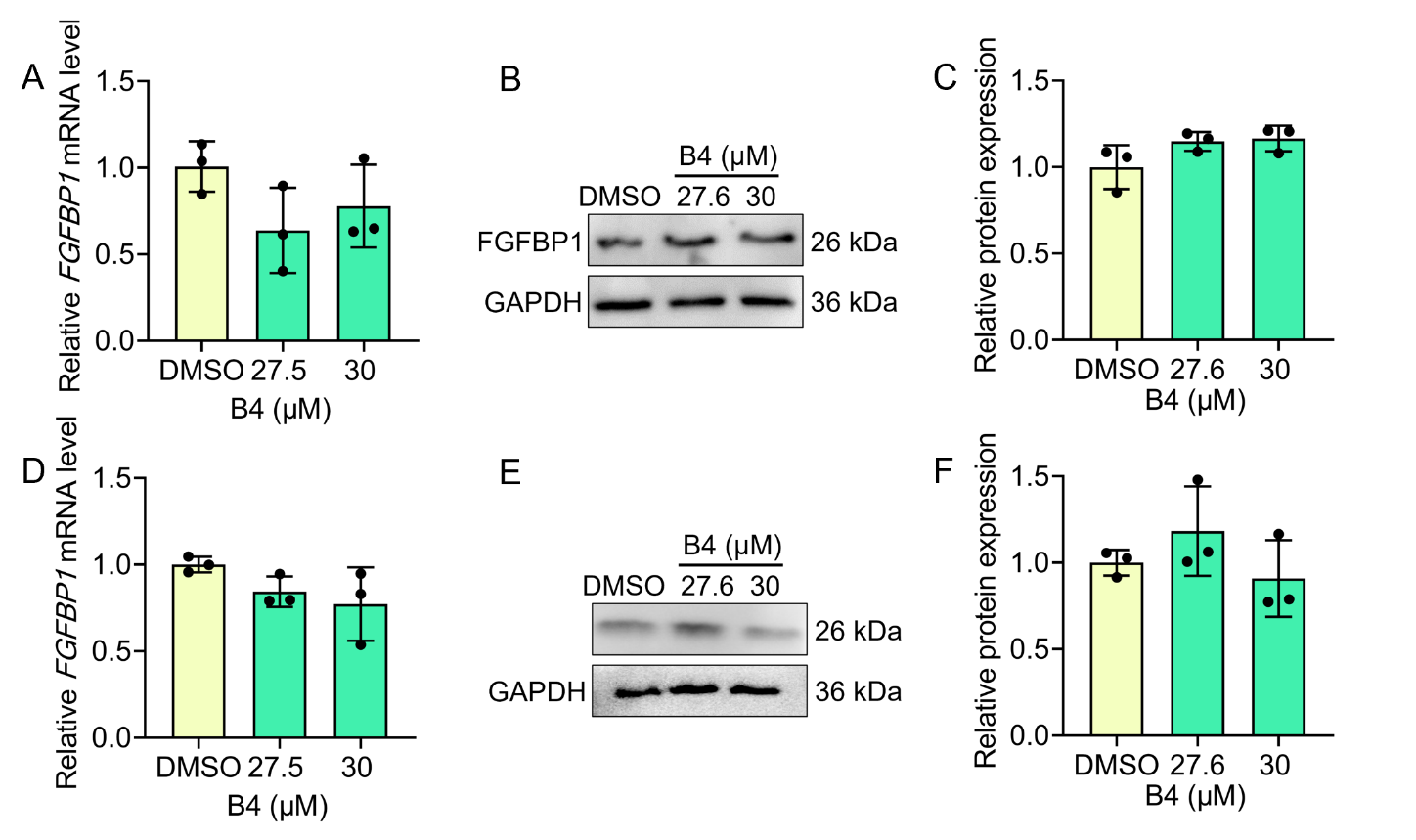


**Supplementary Figure 4** Effect of B4 on HEL and MDA-MB-468 cell lines. (A) RT-qPCR of FGFBP1 in HEL cells. (B, C) Protein expression and densitometry analysis of FGFBP1 after B4 treatment in HEL cells. (D) RT-qPCR of FGFBP1 in MDA-MB-468 cells. (E, F) Protein expression and densitometry analysis of FGFBP1 after B4 treatment in MDA-MB-468 cells.


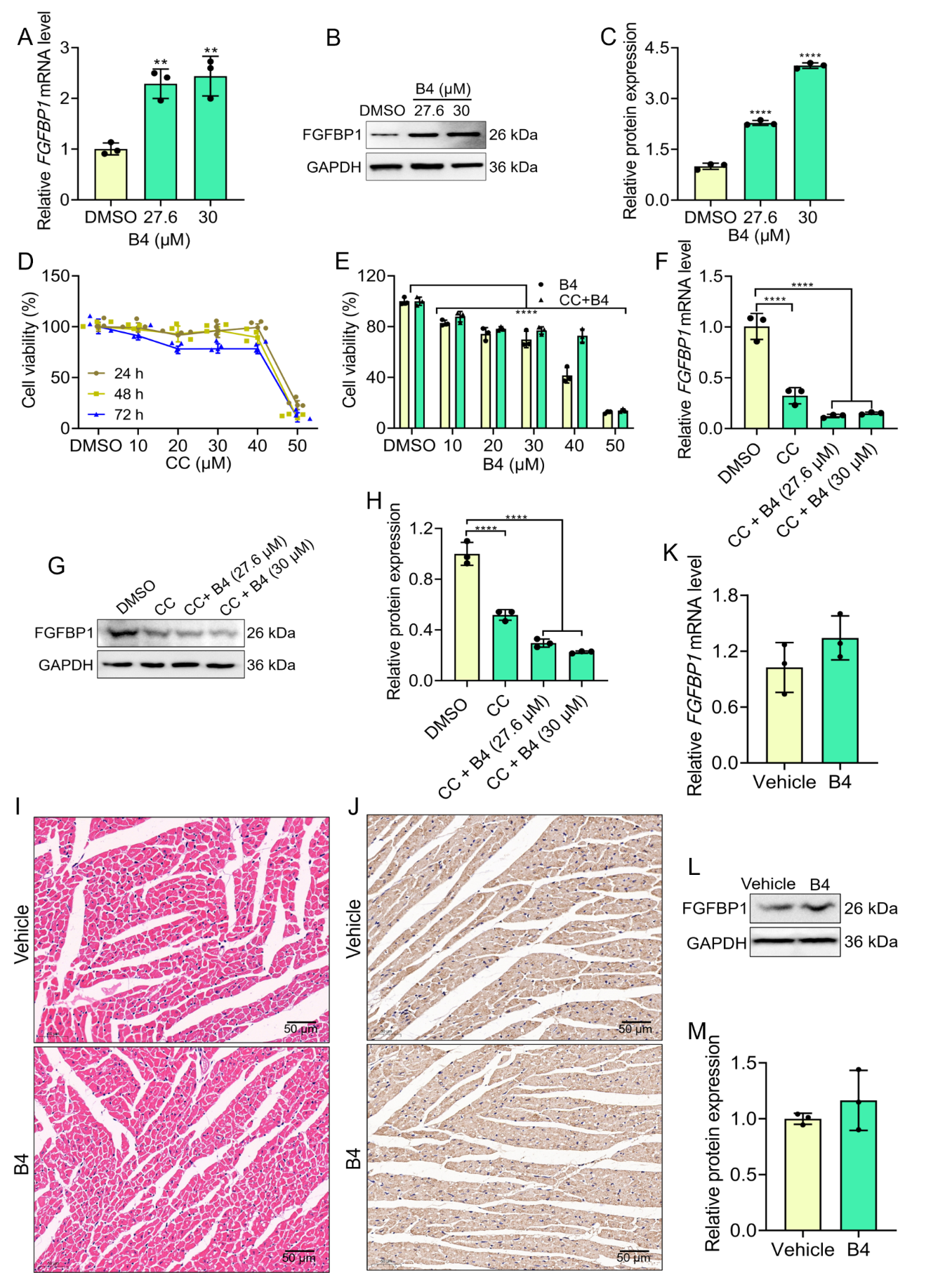


**Supplementary Figure 5** Effect of B4 in vitro and in vivo on heart cells. (A) RT-qPCR analysis of FGFBP1 levels in H9C2 cells after 72 hours of B4 treatment. (B, C) Protein expression and densitometry analysis of FGFBP1 after 72 hours of B4 treatment in H9C2 cells. (D) Dose- and time-dependent cell viability of H9C2 cells treated with B4. (E) Cell viability effect of B4 when treated in combination with CC (10 µM) in H9C2 cells. (F-H) Combinational therapeutic effect on FGFBP1 gene (F) and protein expression (G) levels with densitometry analysis (H) in H9C2 cells. (I) H & E staining of heart tissue from lymphoma vehicle and B4-administered mice. (J) Immunofluorescence of heart tissues from vehicle and B4-treated mice against FGFBP1. (K-M) Gene (K) and protein expression (L) with densitometry analysis (M) of FGFBP1 in heart tissue of vehicle and B4-treated lymphoma mice. Data were represented as mean ± SD (n = 3); ****p < 0.0001, versus as indicated in figures.


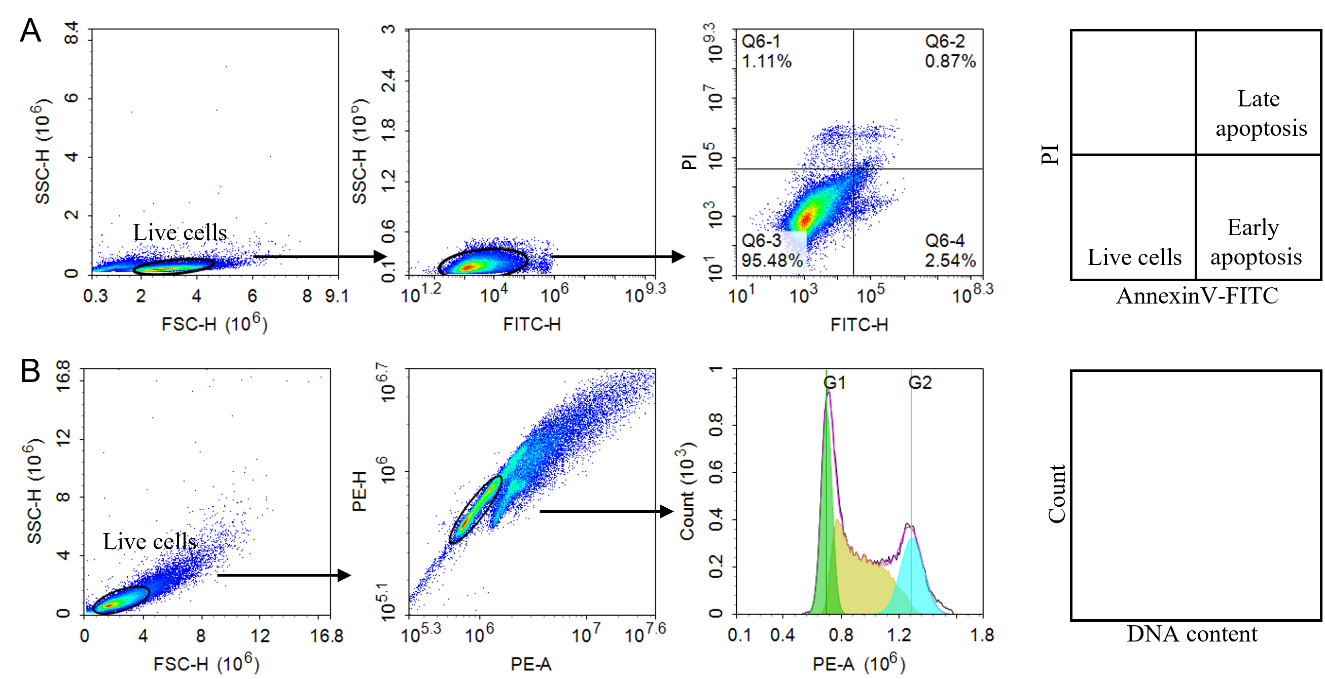


**Flow cytometry gating strategy.** A. Apoptosis analysis using FITC-AnnexinV/PI. 75000 events were captured. B. Cell cycle analysis using PI. The plot shows cells in G0/G1 phase (green), S phase (yellow) and G2/M phase (blue). 50000 events were captured.
